# A Health Belief Model Approach to Determining Psychological Predictors of Behavioral Intention to Seek Health Information on Social Media: Quantitative Descriptive Study

**DOI:** 10.2196/98444

**Published:** 2026-10-22

**Authors:** Sivile Manene, Liezel Cilliers

**Affiliations:** 1 Department of Information Systems, Faculty of Management and Commerce, University of Fort Hare, East London, Eastern Cape, South Africa; 2 Department of Public Health, Faculty of Health Sciences, University of Fort Hare, 50 Church Street, East London, Eastern Cape, 5201, South Africa, 27 0835184009

**Keywords:** misinformation, COVID-19, psychological indicators, risk communication, social media

## Abstract

**Background:**

The increasing use of social media as a source of health information has highlighted the need to understand the psychological factors influencing individuals’ intentions to seek health-related information online, particularly within the South African context.

**Objective:**

This study aimed to determine the psychological predictors of behavioral intention (BI) to seek health information on social media within a developing South African context.

**Methods:**

Adopting a positivist quantitative approach, data were collected from 214 social media users aged 18 to 35 years using a nonprobability convenience sample and analyzed through structural equation modeling using IBM SPSS and SmartPLS.

**Results:**

The findings from the sample of 214 young adults showed that perceived susceptibility (β=0.189; *P*=.01), perceived severity (β=0.288; *P*=.001), health motivation (β=0.462; *P*<.001), perceived benefits (β=0.221; *P*=.02), and cues to action (β=0.281; *P*<.001) significantly increased users’ intentions to seek, verify, and share accurate health information. Self-efficacy had a significant negative effect on BI (β=−0.251; *P*=.008), whereas perceived barriers had no significant effect (β=−0.028, *P*=.65).

**Conclusions:**

Based on these insights, the psychological predictors of BI to seek health information on social media within a South African context are proposed to support users in identifying and managing misinformation, offering practical implications for risk communication strategies and public health interventions.

## Introduction

### Background

The COVID-19 pandemic led to a significant increase in social media usage, with the number of users rising by 10%, amounting to 3.96 billion by the end of June 2020 and further growing to over 4.48 billion users by November 2023 [1,2]. During the early stages of the pandemic, the World Health Organization (WHO) [3] introduced the term “infodemic” to describe the overwhelming spread of both accurate and inaccurate health information, which triggered widespread distrust in health institutions. At the height of the pandemic, social media platforms like Instagram, Facebook, and YouTube experienced a surge of over 620,000 new followers on antivaccination accounts, leading to an influx of misinformation about COVID-19 vaccines and increasing vaccine hesitancy [4]. To mitigate the risks associated with health misinformation, the WHO [5] advised policymakers to implement risk communication and community engagement (RCCE) strategies, which aim to enhance public awareness and influence decision-making regarding health risks. Psychological factors play a crucial role in identifying and addressing risks associated with misinformation, as false narratives can alter beliefs, attitudes, and behaviors, potentially leading individuals to follow unverified medical advice instead of guidance from qualified health professionals [6].

For over 2 centuries, vaccinations have played a crucial role in managing the spread of diseases such as poliomyelitis and smallpox, significantly reducing their prevalence in society [7]. More recently, in the context of COVID-19, vaccines served as a crucial preventative measure against the emergence of new cases and potential variants [3]. However, vaccine hesitancy has been a significant challenge, influenced by factors such as misinformation, distrust in health care systems, individual psychological factors, cultural beliefs, and concerns about safety and efficacy. Vaccine hesitancy refers to one’s unwillingness or reluctance to get vaccinated [8]. To address vaccine hesitancy, the WHO’s [9] Global Advisory Committee on Vaccine Safety (GACVS) developed a COVID-19 vaccine risk communication plan. This plan emphasized the significant influence of social media on individual behavior and recommended that credible information about local situations and misinformation be actively shared on social media platforms. Similarly, South Africa’s Department of Health [10] incorporated mass media campaigns into its RCCE plan, aiming to increase public knowledge and awareness about COVID-19 risks. However, unlike the GACVS’s risk communication plan, South Africa’s RCCE plan does not specifically address the role of social media in the COVID-19 infodemic. To support the development of the RCCE plan, this study aims to determine the psychological predictors of behavioral intention (BI) to seek health information on social media within a South African context.

### Literature Review

Social media has evolved from basic information exchange to sophisticated communication platforms, playing a crucial role in crisis management and information dissemination [11,12]. The South African Medical Research Council [13] reported a surge in social media usage during the COVID-19 pandemic, from 20% to 87% globally. Their analysis revealed that 40% of 112 million posts were unreliable, and nearly 42% of 178 million Twitter (subsequently rebranded X) posts were bot-generated. Literature further suggests that increased social media participation exposes users to false or misleading health information [14]. Yeung et al [15] further argue that the design of popular platforms contributes to the spread of health misinformation, raising concerns about the role of social media in influencing one’s decision-making.

The health belief model (HBM), originally developed in the 1950s to explain individuals’ reluctance to adopt disease prevention behaviors [16], has evolved into a widely applied framework for predicting health behaviors across diverse populations [17]. The model conceptualizes behavior through 2 key dimensions: threat perception, comprising perceived susceptibility (PSU) and perceived severity (PSEV), and behavioral evaluation, comprising perceived benefits (PBEs) and perceived barriers (PBAs) [18]. PSU refers to an individual’s assessment of their vulnerability to a health threat, while PSEV reflects beliefs about the seriousness of its consequences [19,20]. Behavioral evaluation considers the PBEs of taking action against the threat and the PBAs that may hinder such action [20,21].

To address earlier criticism of the model, the HBM was extended to include additional constructs such as health motivation (HM), cues to action (CAs), and self-efficacy [20]. HM reflects an individual’s readiness to engage in behaviors that mitigate perceived threats [22], while CAs refer to internal or external triggers, such as media campaigns or social influence, that prompt behavioral change. Self-efficacy captures an individual’s belief in their ability to perform the required behavior, which is critical in determining whether PBEs translate into action [21]. Collectively, these constructs provide a comprehensive lens through which health behaviors can be understood and influenced.

Within the broader digital context, the HBM is particularly relevant in understanding the impact of health misinformation through social media. The COVID-19 “infodemic” [3] exposed users to vast amounts of misleading information, often shared without malicious intent but driven by engagement incentives such as likes and shares [23,24]. Misinformation has been shown to influence health behaviors negatively, contributing to vaccine hesitancy and reduced adherence to public health measures [25-27]. Social media echo chambers further amplify misinformation, reinforcing distrust in health authorities and shaping attitudes toward vaccination [28,29].

Despite these challenges, social media also presents opportunities for promoting accurate health information through targeted risk communication and situational awareness strategies [30,31]. By applying the HBM within this context, it becomes possible to understand how users perceive risks, evaluate information, and respond to health messaging. This integration of behavioral theory with digital communication environments underscores the model’s continued relevance in addressing contemporary public health challenges, particularly in mitigating the effects of misinformation and improving informed decision-making.

Risk communication is a vital component of crisis management and pandemic preparedness, involving the exchange of information and opinions among individuals, groups, and institutions to manage risks [32]. Since the 1980s, it has evolved as a multidisciplinary field combining public health, psychology, and crisis management to address uncertainty [33]. Effective risk communication strategies include using trusted local health care providers and leveraging social media for public engagement and the promotion of health awareness [34].

Behavioral science theories play a crucial role in facilitating the development and implementation of effective public health interventions. There are two aspects of individuals’ representation of health and health behavior that the HBM focuses on: threat perception, which is construed as PSU and PSEV, as well as behavioral evaluation, which is construed as PBEs and PBAs [18]. PSU refers to the possibility of assigning personal vulnerabilities when developing a particular health condition. This concept assumes that people will more likely behave in healthy ways when faced with the belief that they are vulnerable to adverse health outcomes [20]. PSEV refers to the seriousness of one’s belief in the consequences of exposure to a specific health condition. This concept deals with the idea that an individual will take some form of action toward preventing the potential adverse outcomes of a specific health condition [19]. Leveraging the threat perception aspect of the HBM is crucial in addressing health misinformation, as it highlights the PSEV and susceptibility to health risks, which can motivate individuals to take proactive steps, such as seeking accurate information and accepting the COVID-19 vaccine, thereby mitigating vaccine hesitancy effectively. An international study of college students by Berger et al [32] revealed that PSEV and benefits explained 52% of the variance in vaccination decisions. Based on these findings, the study further recommended that awareness of the severity of vaccine hesitancy, as well as the benefits associated with vaccines, be put forth by stakeholders in order to curb vaccine hesitancy. In another study by Du et al [33], it was also found that lower PSU, higher PBAs, and lower PBEs were associated with COVID-19 vaccine hesitancy.

The following hypotheses were proposed based on the literature:

- H1: PSU to COVID-19 vaccine misinformation does influence users’ intent to seek health information on social media.
- H2: PSEV of COVID-19 vaccine misinformation does influence users’ intent to seek health information on social media.

HM is the individual’s motivation to act on the behavior necessary to mitigate the perceived threat. This concept assumes that the person will act on the behavior based on the perceived susceptibilities, severities, benefits, and barriers regarding the phenomenon [22]. PBEs are the individual’s opinions about the value or usefulness of enacting the necessary behavior to mitigate the perceived threat. This concept measures one’s motivation to act and opt for a precautionary behavior that effectively prevents the threat [21]. Finally, the PBA refers to the individual’s evaluation of the struggles associated with the behavior required to mitigate the perceived threat. The concept assumes that the individual may not perform the necessary behavior despite their belief in its effectiveness against the threat. This is due to the individual’s belief that the likelihood of the threat outweighs the potential benefit [20]. The theory behind behavioral evaluation, based on the HBM, hypothesizes that an individual’s decision to engage in a health behavior is shaped by their evaluation of the benefits and barriers associated with that behavior. This phenomenon is supported by the findings of Du et al [33] and Berger et al [32], which state that perceptions of vaccine benefits drive vaccine uptake, while lower perceived risk, higher barriers, and doubts about the benefits contribute to vaccine hesitancy. From a social media perspective, other research on vaccine hesitancy further highlights the importance of PBEs and PBAs in influencing vaccination attitudes. In a study by Izzati et al [34], the main findings found that PBAs to vaccines, PBEs of vaccines, and knowledge about vaccines were all related to vaccine hesitancy in both antivaccine and provaccine groups on social media.

The following hypotheses are put forward based on the studies cited above:

- H3: HM influences users’ intent to seek health information on social media.
- H4: PBE of the COVID-19 vaccine does influence users’ intent to seek health information on social media.
- H5: PBA of the COVID-19 vaccine does influence users’ intent to seek health information on social media.

As modern times have progressed, the HBM has been refined to address its limitations [20]. The component “CA,” introduced in 1966, refers to internal and external triggers that prompt individuals to engage in behaviors to mitigate perceived threats, including external influences such as mass media and social pressure, as well as internal signals like symptoms [20]. Perceived self-efficacy (PSE), defined as the belief in one’s ability to perform the necessary health behavior, is heavily influenced by trust in health institutions and treatments [35]. Vaccination motivations often stem from trust in science, belief in vaccine efficacy, and the desire to protect oneself and others [36]. Drawing from the literature and prior discussion, effective risk communication and situational awareness are essential for building public trust, reducing misinformation, and promoting informed decisions. Furthermore, self-efficacy plays a critical role in overcoming barriers and recognizing the benefits of health behaviors like vaccination. Weerakoon et al [37] emphasize that trust in health professionals, health literacy, and self-efficacy significantly influence vaccine acceptance, with deficiencies in these areas linked to greater hesitancy. Overall, fostering trust through transparent communication and addressing misinformation is likely to enhance public engagement and health outcomes, ultimately reducing vaccine hesitancy.

The following hypotheses are put forward based on studies cited above:

- H6: PSE toward the COVID-19 vaccine misinformation does influence user’s intent to seek health information on social media.
- H7: CA toward the COVID-19 vaccine influence user’s intent to seek health information on social media.

Figure 1 summarizes the seven hypotheses in a proposed model as explained in the “Literature Review” section.

**Figure 1.**
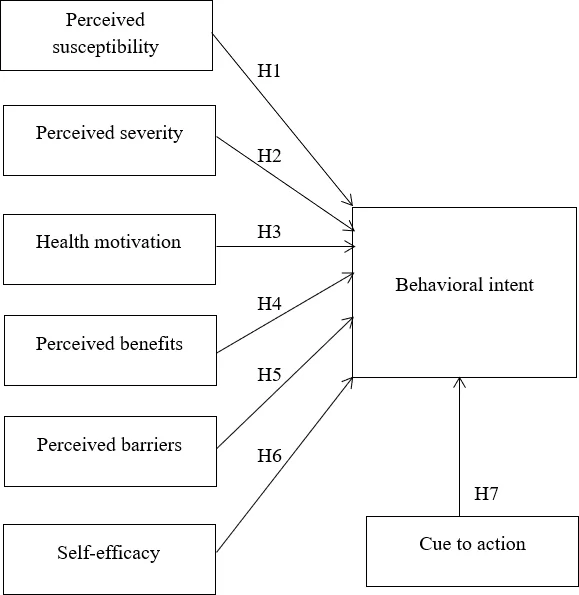
Research framework.

## Methods

### Study Design and Setting

The study adopted a quantitative descriptive research design to determine the psychological predictors to seek health information on social media within a South African context. The target population comprised young adult social-media users aged 18 to 35 years. This population was selected because young adults are among the most active users of social media [38]. Participants were recruited using nonprobability convenience sampling through Facebook and WhatsApp. A sample-size determination table indicated that 384 participants would be appropriate for an estimated population of more than 400,000 young adults residing in the Buffalo City Metropolitan Municipality [39,40].

A total of 214 valid questionnaires were retained for statistical analysis, representing 55.7% of the intended sample of 384. Questionnaires containing missing responses or illogical response patterns, such as selecting the same response for every item, were excluded. Incomplete questionnaires, duplicate submissions, and questionnaires containing illogical response patterns were also excluded. No statistical weighting or other adjustment was applied to account for the nonprobability sampling method.

The questionnaire consisted of 37 items distributed across 6 scales derived from the HBM and 1 scale derived from the theory of reasoned action [18,41-43]. The questionnaire items were adapted from previously published literature and presented in English. Responses were measured using a 4-point Likert-type scale ranging from 1 (“strongly disagree”) to 4 (“strongly agree”; Multimedia Appendix 1).

The questionnaire was pilot tested with 10 participants from the target population to evaluate its clarity, usability, and technical functionality. The pilot testing did not identify a need for questionnaire changes. Pilot participants were excluded from the main study and subsequent statistical analysis.

The final dataset comprised 214 valid questionnaires. Data were analyzed using descriptive statistics and structural equation modeling (SEM). SPSS (IBM Corp) was used for data preparation and descriptive analysis, while SmartPLS 4.0 (SmartPLS GmbH) was used to assess the measurement and structural models.

### Ethical Considerations

Data were collected from March 2025 to June 2025 using a self-administered online questionnaire hosted on SurveyMonkey. An open survey link was advertised and distributed through Facebook and WhatsApp. The questionnaire was presented on a single SurveyMonkey page and required approximately 15 minutes to complete. Participation was voluntary, and no incentives were offered.

Survey data were exported from SurveyMonkey and stored in a password-protected Microsoft OneDrive account. The data will be retained securely for 3 years. Ethical approval was obtained from the Research Ethics Committee (reference: CIL041SMAN01). Before accessing the questionnaire, potential participants received information about the study and provided written electronic informed consent. Participants were informed that participation was voluntary and that they could discontinue the survey before submission. No directly identifying information was reported in the study findings.

## Results

Detailed information on the respondents’ demographics is shown in Table 1. There were 130 female respondents represented in the sample compared to 84 male respondents, and the largest share of the sample, with 115 (53.7%) respondents, was between the ages of 18 and 21 years. Of the 214 respondents, 113 (53%) reported that they lived in an urban area, while 116 (54.2%) indicated that a degree or diploma was their highest level of education.

The data were analyzed by SEM using SmartPLS 4.0. In particular, the analysis focused on using descriptive and inferential statistical techniques. SEM was also implemented to test the relationship between the components of the framework and the underlying variables against which they were measured. As recommended by Hair et al [38], the measurement or “outer” model was analyzed first, followed by the analysis of the structural or “inner” model.

**Table 1.** Demographics of respondents (N=214).

Category	Value, n (%)
Gender	
Male	84 (39)
Female	130 (61)
Age (y)	
18‐21	115 (53.7)
22‐25	86 (40.2)
26‐30	13 (6)
31+	0 (0)
Area of residence	
Urban	113 (53)
Periurban	51 (24)
Informal settlement	9 (4)
Rural	41 (19)
Level of education	
Did not complete high school	2 (0.9)
High school	79 (36)
Higher certificate	16 (7)
Diploma/degree	116 (54.2)
Postgraduate	1 (0.5)

To estimate the appropriateness of the measurement model, Hair et al [38] proposed using the Cronbach α test to determine internal consistency and reliability. Cronbach α values ranged from 0.712 to 0.974, which demonstrated good internal consistency across the constructs. Additionally, the composite reliability values ranged from 0.833 to 0.979, which is well above the recommended threshold of 0.7, further supporting the internal consistency of the measurement scales. The average variance extracted (AVE) values ranged from 0.533 to 0.904, all exceeding the threshold of 0.5, indicating good convergent validity for the constructs. The results presented in Table 2 confirm the reliability and validity of the measurement model, ensuring that the study’s constructs are well-defined and accurately represented.

The internal consistency and convergent validity provide a solid foundation for further analysis, and the relationships identified in the model can be interpreted with confidence. The discriminant validity was assessed using the Fornell-Larcker criterion, with results in Table 3.

**Table 2.** Validity and reliability results.

Subconstruct and items	Factor loadings	CA^a^	CR^b^	AVE^c^
BI^d^		0.794	0.860	0.556
BI1	0.568			
BI2	0.819			
BI3	0.767			
UB1^e^	0.760			
UB2	0.787			
CA		0.719	0.842	0.641
CA1	0.762			
CA2	0.775			
CA3	0.861			
HM^f^		0.745	0.837	0.563
HM1	0.749			
HM2	0.819			
HM3	0.665			
HM4	0.760			
PBA^g^		0.878	0.900	0.564
PBA1	0.835			
PBA2	0.720			
PBA3	0.775			
PBA4	0.773			
PBA5	0.679			
PBA6	0.625			
PBA7	0.825			
PBE^h^		0.893	0.918	0.693
PBE1	0.851			
PBE2	0.780			
PBE3	0.843			
PBE4	0.819			
PBE5	0.865			
PSE^i^		0.934	0.968	0.938
PSE1	0.967			
PSE2	0.970			
PSU^j^		0.886	0.902	0.572
PSU1	0.714			
PSU2	0.884			
PSU3	0.844			
PSU4	0.661			
PSU5	0.813			
PSU6	0.699			
PSU7	0.644			
PSEV^k^		0.727	0.846	0.647
PSEV1	0.858			
PSEV2	0.780			
PSEV3	0.773			

a CA: cue to action.

b CR: composite reliability.

c AVE: average variance extracted.

d BI: behavioral intention.

e UB: usage behavior.

f HM: health motivation.

g PBA: perceived barrier.

h PBE: perceived benefit.

i PSE: perceived self-efficacy.

j PSU: perceived susceptibility.

k PSEV: perceived severity.

**Table 3.** Fornell-Larcker criterion discriminant validity results.^a^

	BI^b^	CA^c^	HM^d^	PBA^e^	PBE^f^	PSE^g^	PSEV^h^	PSU^i^
BI	*0.745*							
CA	0.402	*0.800*						
HM	0.545	0.237	*0.750*					
PBA	0.202	0.331	0.164	*0.751*				
PBE	0.257	0.049	0.067	−0.033	*0.832*			
PSE	0.166	0.029	0.059	−0.027	0.828	*0.969*		
PSEV	0.300	0.001	0.061	−0.023	0.733	0.690	*0.805*	
PSU	0.206	0.092	0.015	0.364	−0.028	−0.037	−0.029	*0.756*

a The number in italics is the square root of average variance extracted (AVE).

b BI: behavioral intention.

c CA: cue to action.

d HM: health motivation.

e PBA: perceived barrier.

f PBE: perceived benefit.

g PSE: perceived self-efficacy.

h PSEV: perceived severity.

i PSU: perceived susceptibility.

Table 3 displays the outcomes of the Fornell-Larcker criterion, which helps assess discriminant validity. This is determined by comparing the square roots of the AVE, shown in bold along the diagonal, with the correlations between latent constructs. According to Hair et al [38], discriminant validity is confirmed when the square root of the AVE for a given construct is greater than the correlations between that construct and others. In this case, the diagonal values, representing the square roots of the AVE, exceed the correlations between constructs for all variables, thereby confirming discriminant validity. For instance, the square root of the AVE for BI is 0.745, which is higher than its correlation with other constructs, such as 0.402 with CA and 0.545 with HM. This pattern holds true across all constructs, supporting the idea that each latent variable is sufficiently distinct from the others.

To provide additional validation of the discriminant validity, the Heterotrait-Monotrait ratio (HTMT) method was also applied, as recommended by Gold et al [39] and Teo et al [40]. The HTMT value should be below or equal to 0.90 to ensure proper discriminant validity. The results of the HTMT test are provided in Table 4, further reinforcing the validity of the model.

**Table 4.** Heterotrait-Monotrait (HTMT) ratio of correlations results.

	BI^a^	CA^b^	HM^c^	PBA^d^	PBE^e^	PSE^f^	PSEV^g^	PSU^h^
BI								
CA	0.533							
HM	0.688	0.311						
PBA	0.210	0.392	0.201					
PBE	0.289	0.189	0.105	0.118				
PSE	0.191	0.140	0.096	0.119	0.847			
PSEV	0.386	0.185	0.101	0.127	0.814	0.839		
PSU	0.194	0.117	0.127	0.436	0.077	0.073	0.103	

a BI: behavioral intention.

b CA: cue to action.

c HM: health motivation.

d PBA: perceived barrier.

e PBE: perceived benefit.

f PSE: perceived self-efficacy.

g PSEV: perceived severity.

h PSU: perceived susceptibility.

Table 4 presents the HTMT ratio of correlations, used to assess discriminant validity. All HTMT values are below the 0.90 threshold recommended by Gold et al [39] and Teo et al [40] confirming that the latent variables are sufficiently distinct from each other. For example, the HTMT value between BI and CA is 0.533, and the highest HTMT value is 0.847 between PSE and PBEs, both of which remain below the critical threshold of 0.90. This supports the discriminant validity of the measurement model, as the latent constructs do not overlap significantly. The results from both the Fornell-Larcker criterion and the HTMT ratio provide strong evidence that the measurement model is valid, with clear differentiation between the constructs.

Before proceeding with hypothesis testing, the model’s fit was thoroughly assessed using several key indices. The Normalized Fit Index was used to determine how well the model reproduced the observed covariance matrix in comparison to a null model, with values exceeding 0.90 considered indicative of an acceptable fit, following the guidelines developed by Bentler and Bonett [41] and Byrne [43].

Moreover, the standardized root mean square residual (SRMR) was used to evaluate the discrepancy between the observed covariance matrix and the one implied by the model. In line with recommendations from Hu and Bentler [42], an SRMR value below 0.08 was deemed appropriate for partial least squares path modeling. The SRMR was found to be 0.078, which demonstrates that the proposed model adequately represents the relationships between the variables.

In this study, SmartPLS was used to analyze both observable and latent variables, focusing on the path coefficients representing their relationships. These coefficients provide essential insights into the strength and direction of the connections among the variables being studied. The hypotheses were analyzed using SmartPLS, and the results are presented in Table 5.

**Table 5.** Result of the structural "inner" model.

Hypothesis	Relationship	Path coefficient	SE	*t* test^a^	*P* values	Decision
H1	PSU^b^→BI^c^	0.189	0.073	2.587	.01	Supported
H2	PSEV^d^→BI	0.288	0.084	3.411	.001	Supported
H3	HM^e^→BI	0.462	0.057	8.045	<.001	Supported
H4	PBE^f^→BI	0.221	0.095	2.335	.02	Supported
H5	PBA^g^→BI	−0.028	0.062	0.458	.65	Not supported
H6	PSE^h^→BI	−0.251	0.094	2.670	.008	Supported
H7	CA^i^→BI	0.281	0.057	4.943	<.001	Supported

a The significance of the structural path coefficients was assessed using a nonparametric bootstrapping procedure in SmartPLS. Because bootstrapping was used, conventional degrees of freedom were not specified for the individual t-statistics. Hypothesis testing was conducted using a 2-tailed test at the 5% significance level..

b PSU: perceived susceptibility.

c BI: behavioral intention.

d PSEV: perceived severity.

e HM: health motivation.

f PBE: perceived benefit.

g PBA: perceived barrier.

h PSE: perceived self-efficacy.

i CA: cue to action.

The structural model was assessed by calculating the coefficients of *R*^2^ and the significance of path coefficients as suggested by Hair et al [38]. The path coefficients ranged from −0.251 to 0.462, indicating sufficient explanatory power of the proposed model. The hypothesized relationships were tested by running a bootstrapping procedure to estimate the path coefficients’ significance. As shown in Table 5, BI was positively and significantly influenced by PSU, PSEV, HM, PBE, PSE, and CA. These results support H1-4 and H7, respectively. PSE was negatively and significantly associated with BI, supporting H6. Only H5 was not supported in this model, as PBA was found not to be significantly associated with BI. Based on these findings, it is noteworthy that a substantial portion, precisely 49.9%, of the total variability observed in BI is attributable to PSU, PSEV, HM, PBE, PBA, PSE, and CA. These results signify that the model comprehensively explains the dynamics influencing social media users’ behavioral intent to seek health information on social media.

## Discussion

### Principal Findings

This study aimed to determine the psychological predictors of BI to mitigate health misinformation on social media within a South African context. The results showed that PSU, PSEV, HM, PBE, and CA significantly increased users’ intentions to seek, verify, and share accurate health information. PBA had no significant effect, while PSE had a significant negative effect, suggesting that users who felt confident in identifying misinformation were less likely to engage in corrective actions.

The results of the SEM, as depicted in Table 5, indicate that PSU had a positive effect on BI, with the β coefficient equaling 0.189 and *P*=.01. According to the theoretical underpinnings of Rosenstock’s HBM [44], individuals who perceive a higher risk of exposure to misinformation are more motivated to take preventive actions. In this study, the results demonstrated that as the users’ awareness of their vulnerability to false information increases, so does their motivation to find accurate health information on social media.

In a similar manner to PSU, PSEV plays a critical role in shaping one’s behavior in preventing health risks. The hypothesis that PSEV of COVID-19 vaccine misinformation influences users’ intent to seek health information on social media was established on the supposition that one would naturally view a particular health risk in terms of its potential consequences [44]. These theoretical foundations suggest that individuals’ actions are heavily influenced by their perceptions of how consequences may affect their health. The results in this study demonstrated that PSEV had a significant and positive effect on BI, with a β coefficient of 0.288 and *P*=.001. This indicates that when social media users perceive the consequences of misinformation about the COVID-19 vaccine as severe, they are more likely to actively seek accurate health information on social media platforms. These findings suggest that when users recognize the serious health and societal impacts of misinformation, they are more motivated to engage with credible information. This aligns with the HBM’s theoretical stance, which posits that higher PSEV can lead to increased health-protective behaviors.

Considering the role of misinformation in fostering mistrust, a high perception of potential consequences might theoretically lead to increased acceptance of preventive measures, further emphasizing the importance of psychological predictors in mitigating the risks associated with this type of information disorder. The concept of HM involves an individual’s interest in maintaining or improving their health. It reflects a proactive attitude toward health and wellness and can influence how a person responds to perceived threats and benefits related to health behaviors. In this particular study, the results indicated that HM significantly influences BI concerning engagement with COVID-19 vaccine information and the mitigation of misinformation on social media. The strong positive path β coefficient of 0.462 and the statistically significant *P*<.001 demonstrated a robust relationship, suggesting that individuals with higher HM are more inclined to adopt proactive behaviors regarding vaccine-related health information. This aligns with the theoretical underpinnings of the HBM, where individuals with high HM perceive a greater benefit in engaging with health-related actions, thereby enhancing their BI. Furthermore, motivated individuals are likely to use cognitive strategies to assess information credibility and may serve as influencers within their social networks by promoting accurate health messages.

Unlike PSU and PSEV, which measure threat perception, PBE and PBA are geared toward assessing behavioral evaluation among individuals. The hypothesis that the PBE of the COVID-19 vaccine influences social media users’ intent to seek health information on social media stems from the HBM’s theoretical premise, which postulates that individuals will take action based on the benefits they perceive in different health options [44]. In the context of this study, individuals who seek health information on social media and engage with accurate vaccine information are more motivated to act, whether by fact-checking, sharing credible sources, or participating in digital discussions on vaccine safety and efficacy. This is reflected in the results of this study, which demonstrated that PBE played a crucial role in shaping BI concerning the participant’s engagement with COVID-19 vaccine information. A positive path β coefficient of 0.221 and a statistically significant *P*=.02 confirmed that individuals recognized the benefits of engaging with health information on social media.

While PBE significantly influences BI, it is important to consider potential barriers to engagement. Within the theoretical supposition of the HBM, Rosenstock’s [44] argued that the actions toward a particular health behavior may be inconvenient, expensive, unpleasant, painful, or even upsetting, resulting in refusal to take action toward said behavior. This is what the theory refers to as the individuals’ PBA. In the context of COVID-19 vaccination, someone might recognize the severity of COVID-19 and believe the vaccine is beneficial but might not get vaccinated due to PBA like misinformation, fear of side effects, or even lack of access to vaccination sites. Based on the given outline of the theoretical premise of this component, this study hypothesized that PBA of the COVID-19 vaccine will influence the user’s intent to seek health information on social media. The results of the study indicated that PBAs in fact do not play a significant role in determining whether individuals engage in seeking health information about the COVID-19 vaccine. The small and statistically insignificant path β coefficient of −0.028 combined with a high *P*=.65 suggests that barriers to engagement are not a decisive factor influencing users’ intent to act. Consequently, this indicates a failure to reject the null hypothesis (H0_5_), meaning that PBAs do not significantly influence BI. One possible explanation for this discrepancy is that users who are motivated to engage with health information may perceive the benefits of engagement as outweighing any potential obstacles. This aligns with the theory that individuals with high HM (as seen in H3) are less likely to be influenced by PBAs because their commitment to accurate health information overrides concerns about challenges. However, while PBA did not show a significant direct effect, it is still crucial for health communicators and policymakers to identify and address potential engagement obstacles. Even if barriers do not significantly affect overall intent, they may still impact specific subgroups, such as individuals with lower digital literacy or those in communities where misinformation is deeply embedded in social structures.

PSE is another added component of the established model which serves as a means for measuring one’s ability to successfully perform a behavior that leads to a desired outcome. This component was crucial in determining whether individuals will adhere to the preventative measures about COVID-19 vaccine misinformation such as those suggested by various RCCE strategies like the established psychological predictors. The term self-efficacy refers to one’s belief about their capabilities to produce a certain level of performance that could influence events in their lives [45]. In this case, PSE was measured based on the premise that, if the individual were to show a positive correlation toward this component, it may negatively influence their BI toward seeking health information about the COVID-19 vaccine. Hence the established hypothesis which states that PSE toward the COVID-19 vaccine misinformation does influence the user’s intent to seek health information on social media. The results of this measurement revealed that PSE had a negative effect on BI with path β coefficient of −0.251 and a *P*=.008. Contrary to the conventional HBM assumption that greater self-efficacy strengthens BI, the significant negative relationship observed in this study may indicate that high confidence does not always translate into greater motivation to act. Participants who considered themselves capable of identifying or managing health misinformation may have felt less need to seek additional information, verify claims, or adopt the proposed behavior. This could reflect overconfidence; whereby perceived competence exceeds actual ability and reduces the effort or cognitive resources allocated to a task. In an online health context, confident individuals may also rely on existing knowledge or personal judgment rather than actively consulting credible information sources. The finding may therefore represent a motivational saturation or complacency effect, rather than evidence that self-efficacy is inherently detrimental. Further research should distinguish accurately calibrated self-efficacy from overconfidence and examine possible nonlinear relationships between self-efficacy and BI. The hypothesis that CA toward the COVID-19 vaccine influences the user’s intent to seek health information on social media stems from Rosenstock’s [44] supposition which recognized that, even if the individual perceives a health threat and sees the benefits of taking action, they may still not act unless they’re prompted by a CA. The findings indicate that CA had a positive effect on BI with a statistically significant path β coefficient of 0.281 and a *P*<.001. This finding highlights the critical role of internal and external stimuli in motivating individuals to engage with COVID-19 vaccine misinformation. The significant positive effect suggests that when individuals receive clear and compelling cues, they are more likely to take proactive steps to seek health information on social media.

Figure 2 below is the final framework contribution developed for this study. The arrows indicate a relationship between each of the components of the framework and the control variable BI, with dark arrows indicating the accepted hypotheses and the dotted arrow indicating the rejected hypothesis.

**Figure 2.**
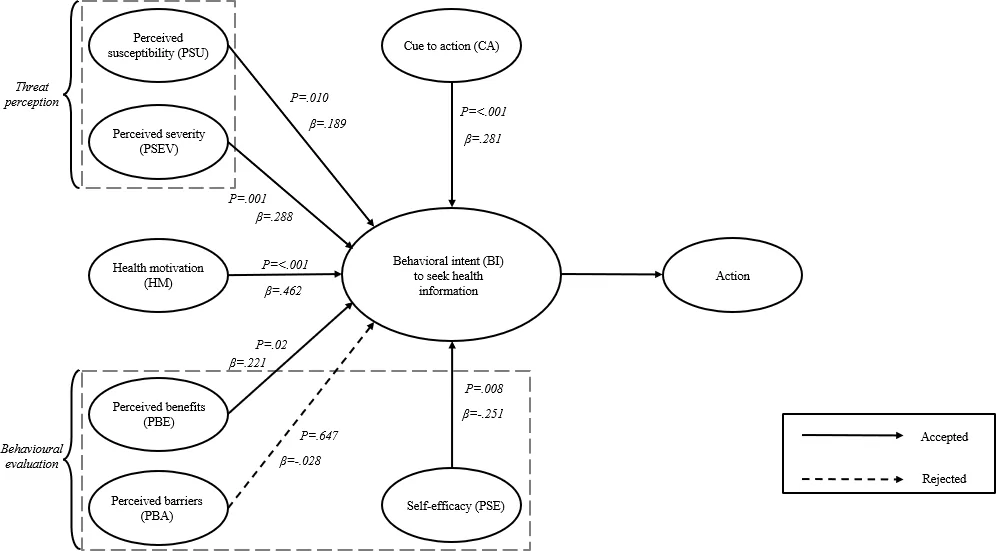
Framework for mitigating health misinformation.

### Practical and Policy Implications

Guided by the theoretical underpinnings of the HBM, the established psychological predictors can have several significant real-world applications, particularly in social media management and RCCE. Since PBAs were rejected by the study’s hypothesis testing, the focus is likely to be on other key aspects of the HBM, such as PSU, PSEV, PBE, HM, and CA.

The identified psychological predictors for mitigating health misinformation on social media offer a tool for public health authorities and social media platforms to use for public health communication and awareness campaigns. When it comes to strengthening users’ threat perception, the framework can target PSU and PSEV by understanding how individuals perceive their risk of possible health complications and the potential severity of the complications. Health communicators can craft messages that emphasize these factors by using credible sources of health information. This could help in countering the risks associated with misinformation, which downplays the severity of a potential health crisis or the effectiveness of preventative measures.

When it comes to the framework’s policy implementation, the results of the study can help policymakers understand the role of individual psychological predictors in shaping public perceptions of preventative measures in the event of a health crisis. Policymakers could leverage the framework to develop awareness campaigns geared toward strengthening threat perception, fostering behavioral evaluation, and increasing triggers that would influence users toward the necessary health behavior.

### Limitations and Recommendations for Future Research

The study is subject to limitations. The study only focused on social media users between the ages of 18 and 35 years living in the Buffalo City Metropolitan area in the Eastern Cape province in South Africa. While the age range is appropriate for social media use, it might exclude important perspectives from older adults who also engage with social media. Furthermore, while conducting the study within the South African environment is important, given the unique sociopolitical, cultural, and health care context, a discussion on how the findings can be generalized or applied in other African or global contexts could be beneficial. Given the limitations outlined, future research should expand the demographic scope to include older adults and diverse geographic locations, providing a more comprehensive understanding of misinformation dynamics. The framework should also be validated in various contexts, including different types of information disorder and different social media platforms.

## Supplementary material

10.2196/98444 Multimedia Appendix 1 Questionnaire used in study.

## Abbreviations

AVE: average variance extracted
BI: behavioral intention
CA: cue to action
GACVS: Global Advisory Committee on Vaccine Safety
HBM: health belief model
HM: health motivation
HTMT: Heterotrait-Monotrait ratio
PBA: perceived barrier
PBE: perceived benefit
PSE: perceived self-efficacy
PSEV: perceived severity
PSU: perceived susceptibility
RCCE: risk communication and community engagement
SEM: structural equation modeling
SRMR: standardized root mean square residual
WHO: World Health Organization
