# Supplementary material for: A Health Belief Model Approach to Determining Psychological Predictors of Behavioral Intention to Seek Health Information on Social Media: Quantitative Descriptive Study
Source: Online J Public Health Inform. 2026 Oct 22;18:e98444. doi: 10.2196/98444 (PMC13690338; doi:10.2196/98444)
Supplement: Supplementary file 1 [file ojphi-v18-e98444-s001.docx]

Demographic Information					
1. Gender:	Male	Female	Neutral		
2. Age:	18-21	22-25	26-30	31+	
3. Area of residence:	Urban	Peri Urban	Informal settlement	Rural	
4. Level of education:	Below Matric	Matric	Higher Certificate	Diploma/degree	Post Grad

I would like to ask you for your opinion and about your experience using social media and the internet for health information. For each statement, tell me which response best reflects your opinion and experience *right now*.

No	Please put a cross in the appropriate block indicating whether you strongly disagree, disagree, neither agree nor disagree, agree or strongly agree with each of the following statements:	Strongly disagree	Disagree	Agree	Strongly agree
1.	Misinformation about the COVID-19 vaccine will likely get worse in the future.	1	2	3	4
2.	It is possible that misinformation on social media could change my opinions about the COVID-19 vaccine.	1	2	3	4
3.	I am likely to encounter vaccine misinformation on social media.	1	2	3	4
4.	I trust the information about the COVID-19 vaccine that I come across on social media.	1	2	3	4
5.	I will likely come across misinformation about the COVID-19 vaccine while using social media.	1	2	3	4
6.	I believe I have control over avoiding exposure to misinformation about the COVID-19 vaccine social media.	1	2	3	4
7.	I have personally encountered and/or witnessed instances of misinformation about the COVID-19 vaccine on social media.	1	2	3	4
8.	I believe misinformation about the COVID-19 vaccine on social media could impact my own personal health.	1	2	3	4
9.	I am aware of the potential consequences associated with misinformation about the COVID-19 vaccine on social media.	1	2	3	4
10.	I believe misinformation about the COVID-19 vaccine on social media has influence on an individuals' decision-making.	1	2	3	4
11.	The benefits of the COVID-19 vaccine outweigh the costs	1	2	3	4
12.	I am more likely to get vaccinated if my healthcare provider recommends it.	1	2	3	4
13.	I have enough information to make an informed decision about getting vaccinated.	1	2	3	4
14.	I trust government health agencies to provide accurate information about the COVID-19 vaccines.	1	2	3	4
